# Supplementary material for: Single-cell expression profiling of bat wing development
Source: Nat Commun. 2025 Jul 18;16:6612. doi: 10.1038/s41467-025-61944-2 (PMC12274464; doi:10.1038/s41467-025-61944-2)
Supplement: Supplementary file 1 — Supplementary Information [file 41467_2025_61944_MOESM1_ESM.pdf]

Supplementary Information

Single-cell expression profiling of bat wing development

Xue Lyu[1,4], Jing Bai[1,2,4], Ji-Bin Jiang[1,2,4], Chang-Jie Sun[1,2], Peng Chen[1], Qi Liu[1], Yuan-Shuo Ma[1,2], and Zhen Liu[1,2,3*]

[1]State Key Laboratory of Genetic Evolution & Animal Models, Kunming Institute of Zoology, Chinese Academy of Sciences, Kunming, China

[2]University of Chinese Academy of Sciences, Beijing, China

[3]Yunnan Key Laboratory of Biodiversity Information, Kunming, China

[4]These authors contributed equally: Xue Lyu, Jing Bai, Ji-Bin Jiang

[*]Correspondence to:

Zhen Liu
Email: zhenliu@mail.kiz.ac.cn
State Key Laboratory of Genetic Evolution & Animal Models
Kunming Institute of Zoology, Chinese Academy of Sciences
Kunming 650201, Yunnan, China
Phone: 86-0871-65199318
Fax: 86-0871-65199318

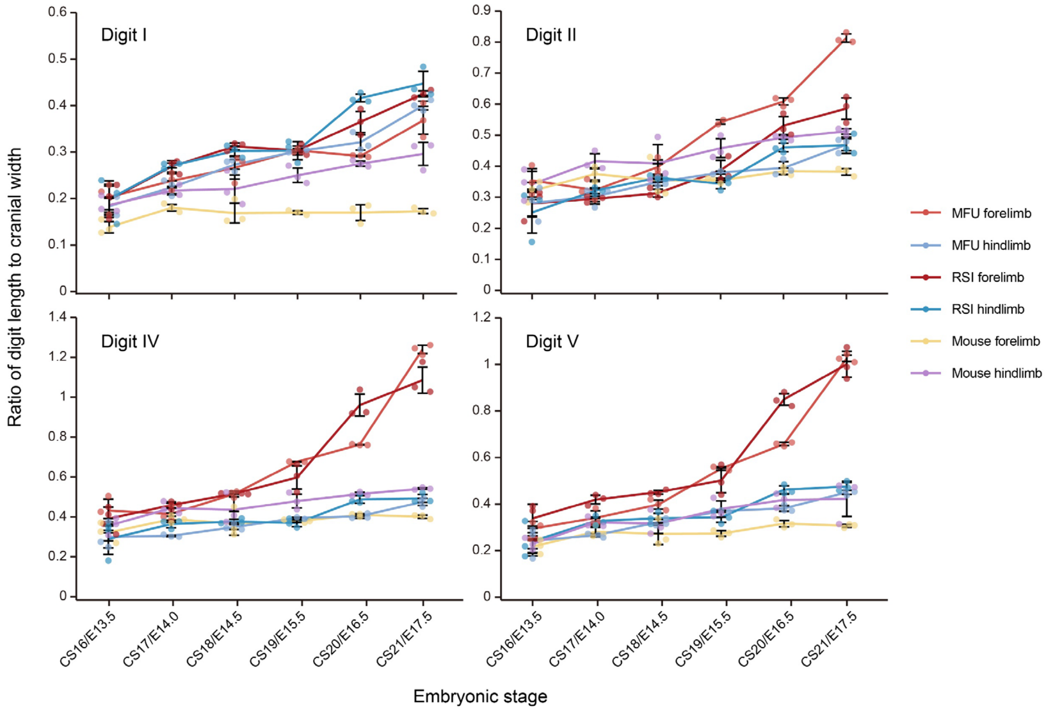

Supplementary Fig. 1. The ratios of the lengths of digits I, II, IV and V to cranial width across six developmental stages in the forelimbs and hindlimbs of two bat species and laboratory mice. RSI and MFU represent the bat species *Rhinolophus sinicus* and *Miniopterus fuliginosus*. All data were presented as mean ± SD in error bars, three individuals per stage per species were used, except for MFU at CS19 with only two individuals.

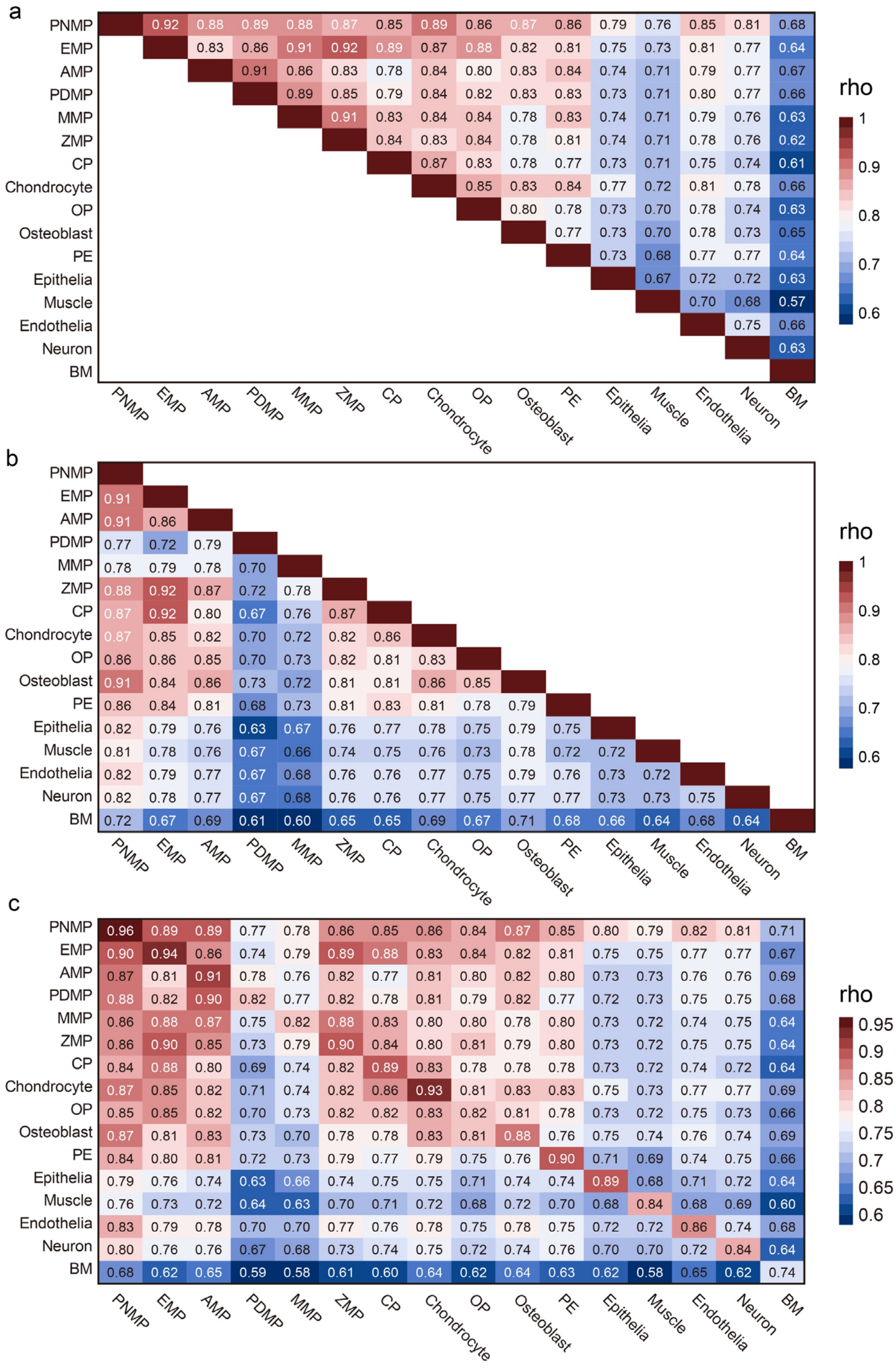

Supplementary Fig. 2. Expression correlations among different cell populations for the top 2,000 variable genes. The correlations are shown for developing forelimbs (a), hindlimbs (b), and between forelimbs and hindlimbs (c).

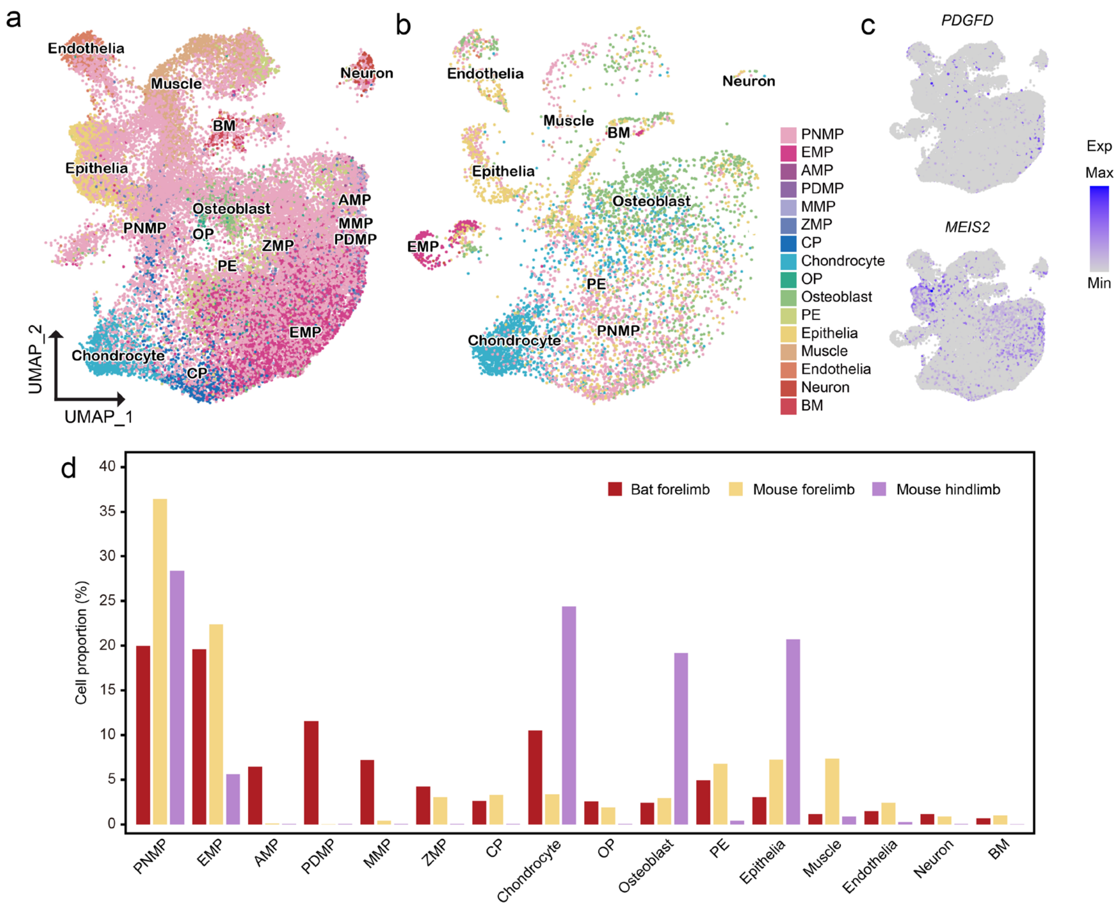

Supplementary Fig. 3. UMAP visualization of color-coded cell populations in developing mouse forelimbs (a) and hindlimbs (b), using labels transferred from the bat scRNA-seq dataset. (c) Expression patterns of the marker genes *PDGFD* and *MEIS2* in mouse scRNA-seq datasets. (d) Cell proportions across bat forelimbs, mouse forelimbs, and mouse hindlimbs.

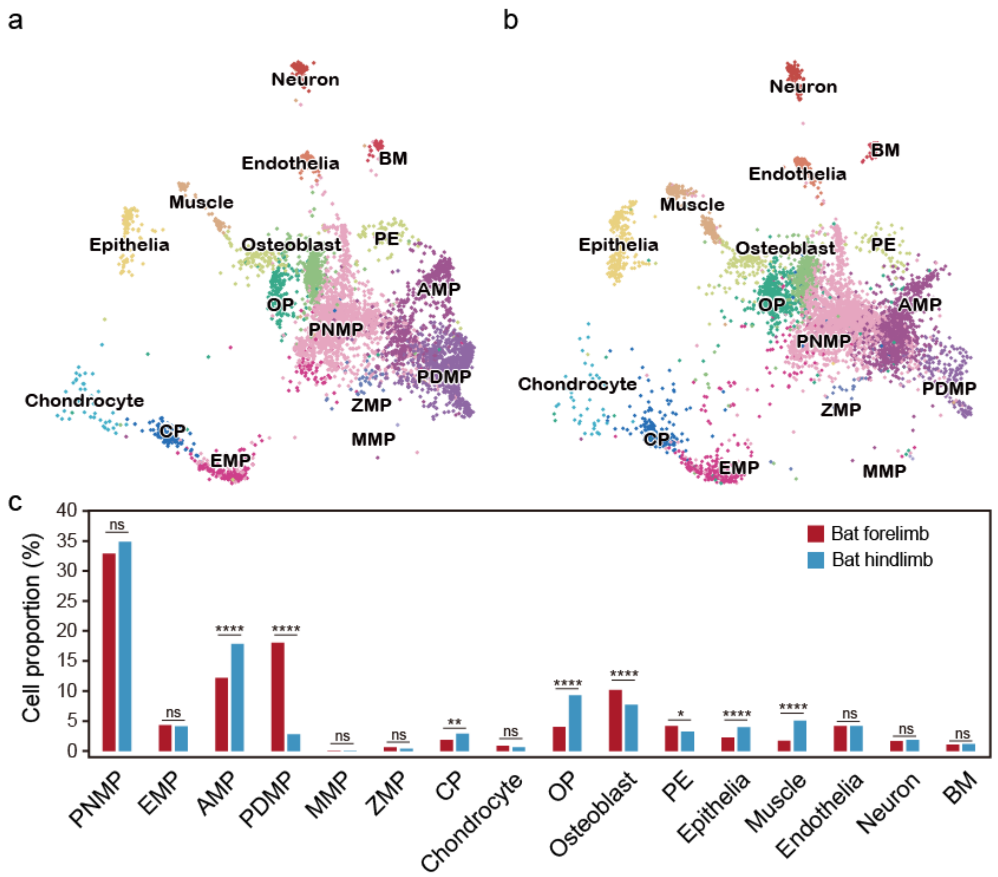

Supplementary Fig. 4. UMAP visualization of color-coded cell populations in developing bat forelimbs (a) and hindlimbs (b) dervied from 10× Genomics datasets, with labels transferred from bat SPLiT-seq datasets. (c) Comparison of cell proportions across various cell populations between developing bat forelimbs and hindlimbs based on 10× Genomics datasets. Statistical significance is determined using χ2 tests. **P* < 0.05; ***P* < 0.01; ****P* < 0.001; *****P* < 0.0001; ns: not significant.

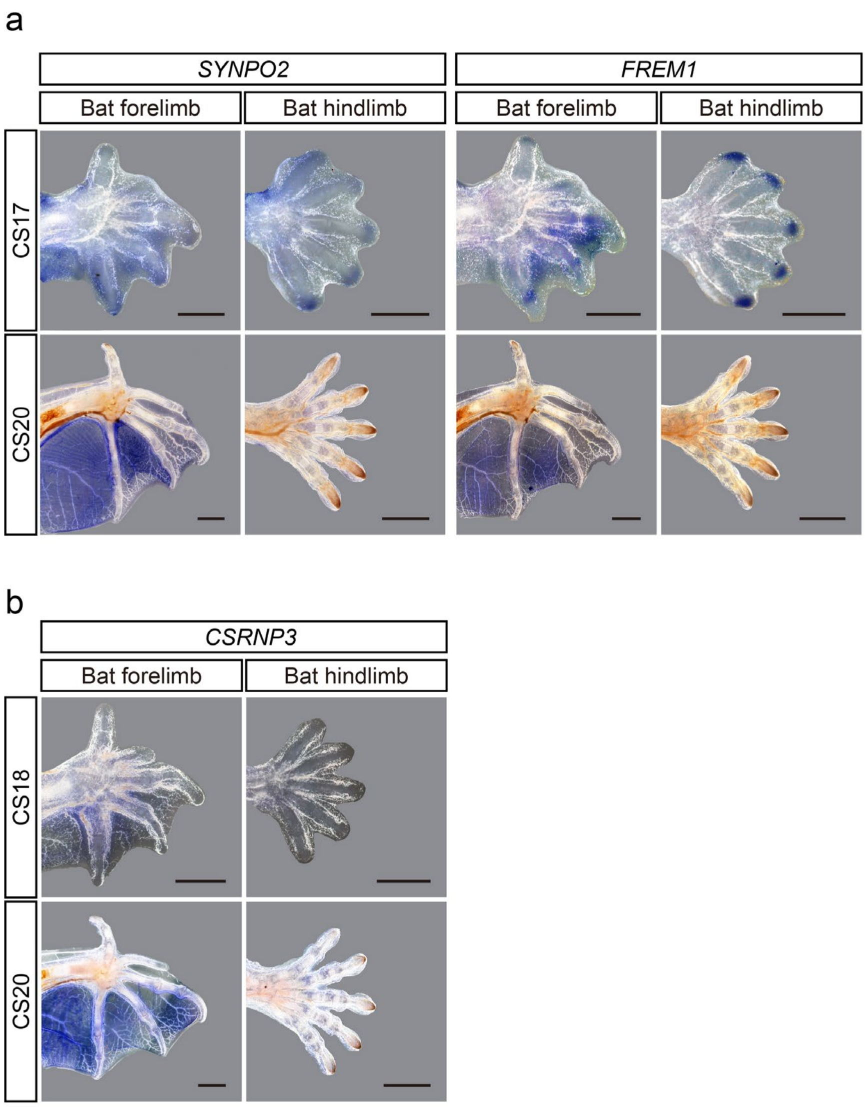

Supplementary Fig. 5. *In situ* hybridization for the marker genes *SYNPO2* and *FREM1* for MMPs (a) and *CSRNP3* for PDMPs (b) in bat forelimbs and hindlimbs at stages CS17/CS18 and CS20. Scale bars: 1 mm.

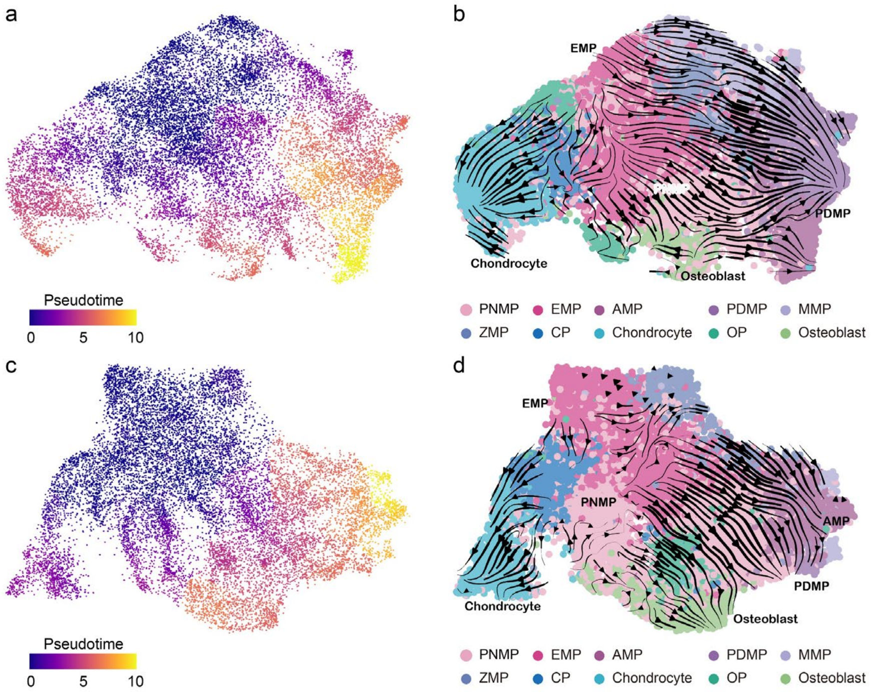

Supplementary Fig. 6. Pseudo-temporal developmental dynamics of mesenchymal and osteochondral cells, along with PNMPs, are depicted for bat forelimbs (a and b) and hindlimbs (c and d) utilizing Monocle3 and scVelo.

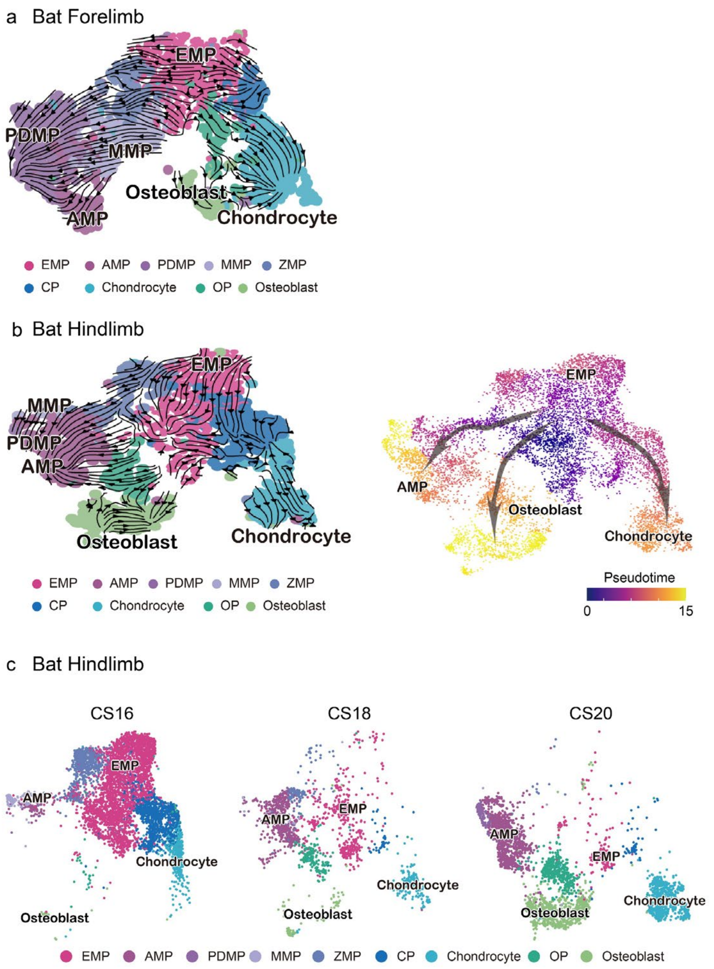

Supplementary Fig. 7. (a) RNA velocity dynamics of mesenchymal and osteochondral cells without PNMPs in developing bat forelimbs. (b) Single-cell trajectories of mesenchymal and osteochondral cell populations in developing bat hindlimbs, with arrows indicating predicted developmental paths. (c) The developmental dynamics of mesenchymal and osteochondral cell populations in developing bat hindlimbs.

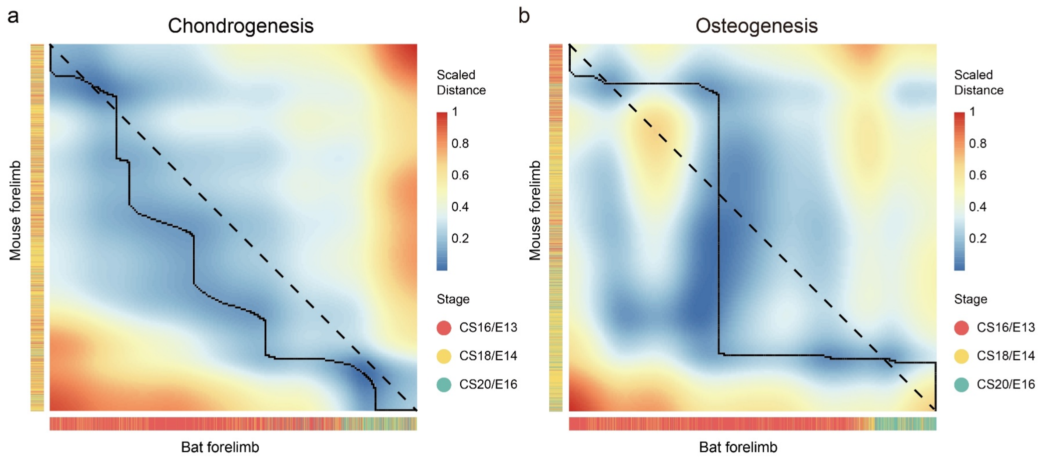

Supplementary Fig. 8. Dissimilarity matrix generated from comparative global alignments of gene expression modules along the chondrogenesis (a) and Osteogenesis (b) trajectories between developing bat forelimbs and mouse forelimbs.

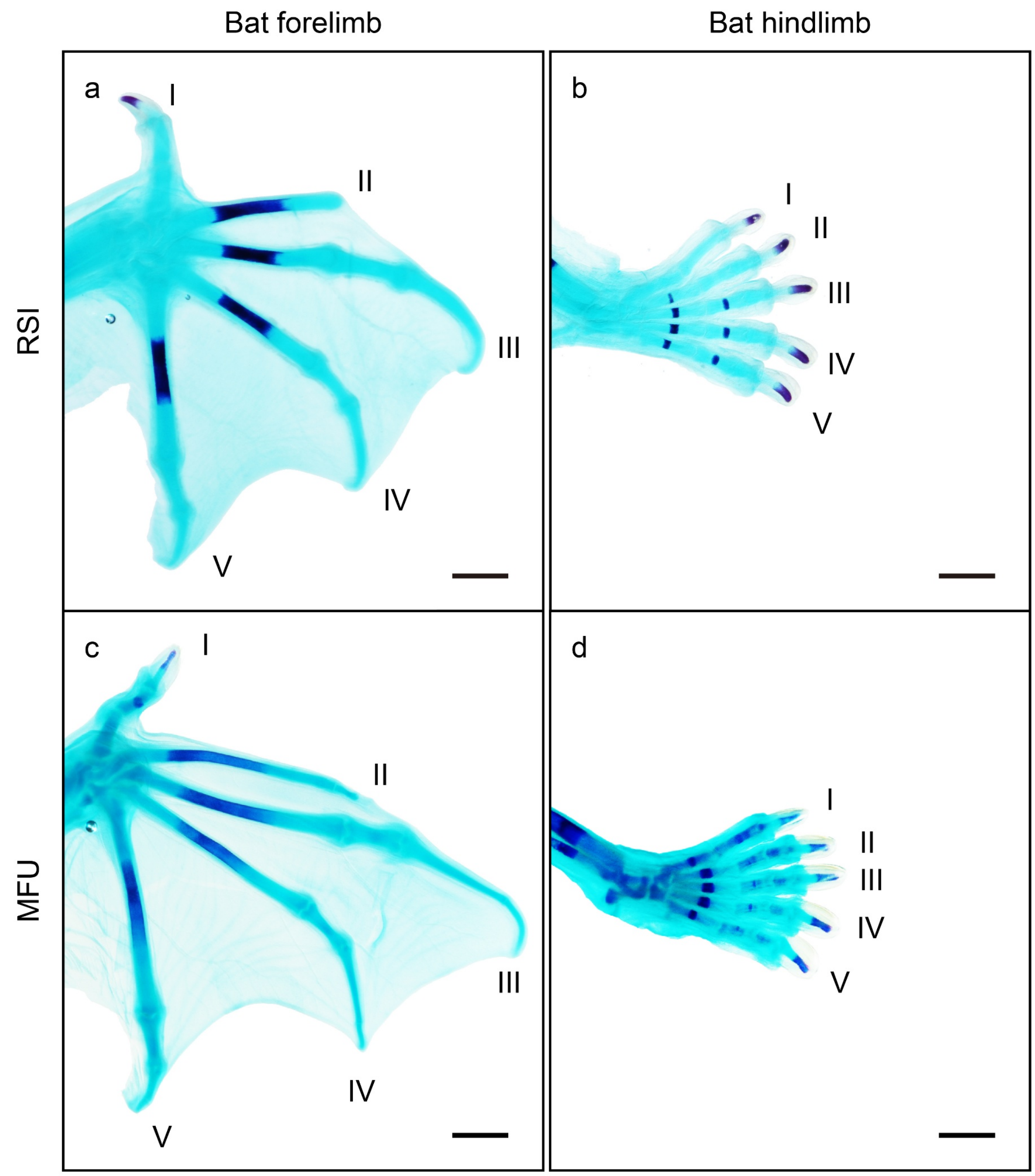

Supplementary Fig. 9. Alcian blue and alizarin red staining of forelimbs and hindlimbs at CS20 for the Chinese horseshoe bat (*Rhinolophus sinicus*, RSI, panels a & b) and the eastern bent-winged bat (*Miniopterus fuliginosus*, MFU, panels c & d). Scale bar: 1 mm.

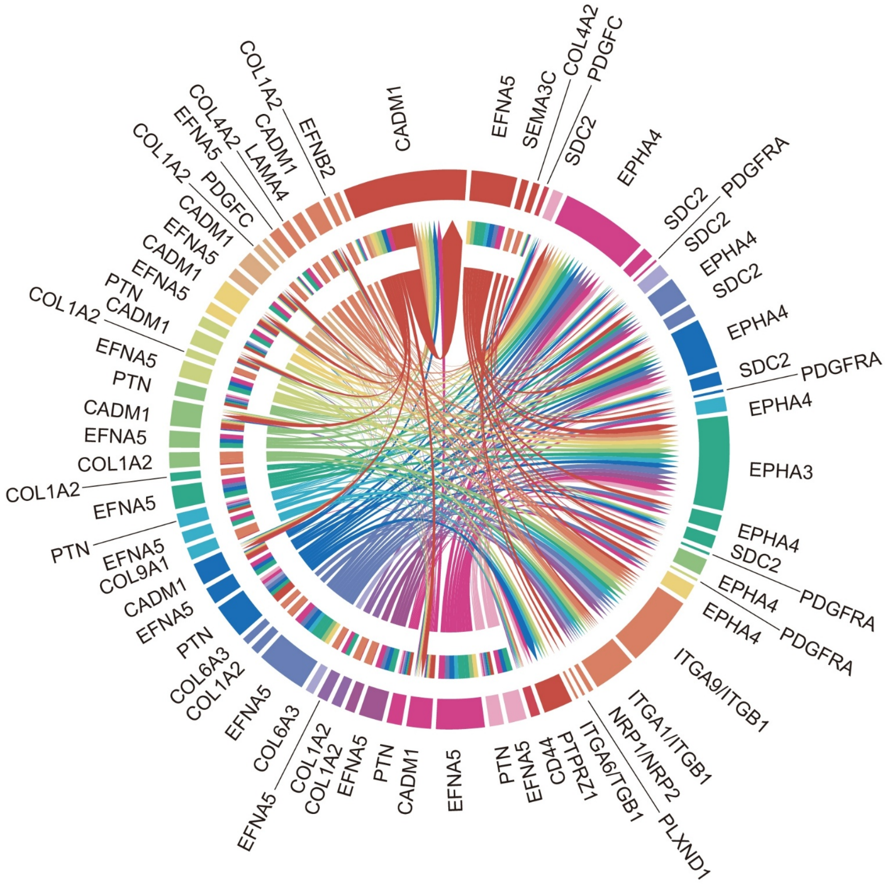

Supplementary Fig. 10. Overview of the up-regulated signaling pathway networks during the development of bat forelimbs compared to mouse forelimbs.

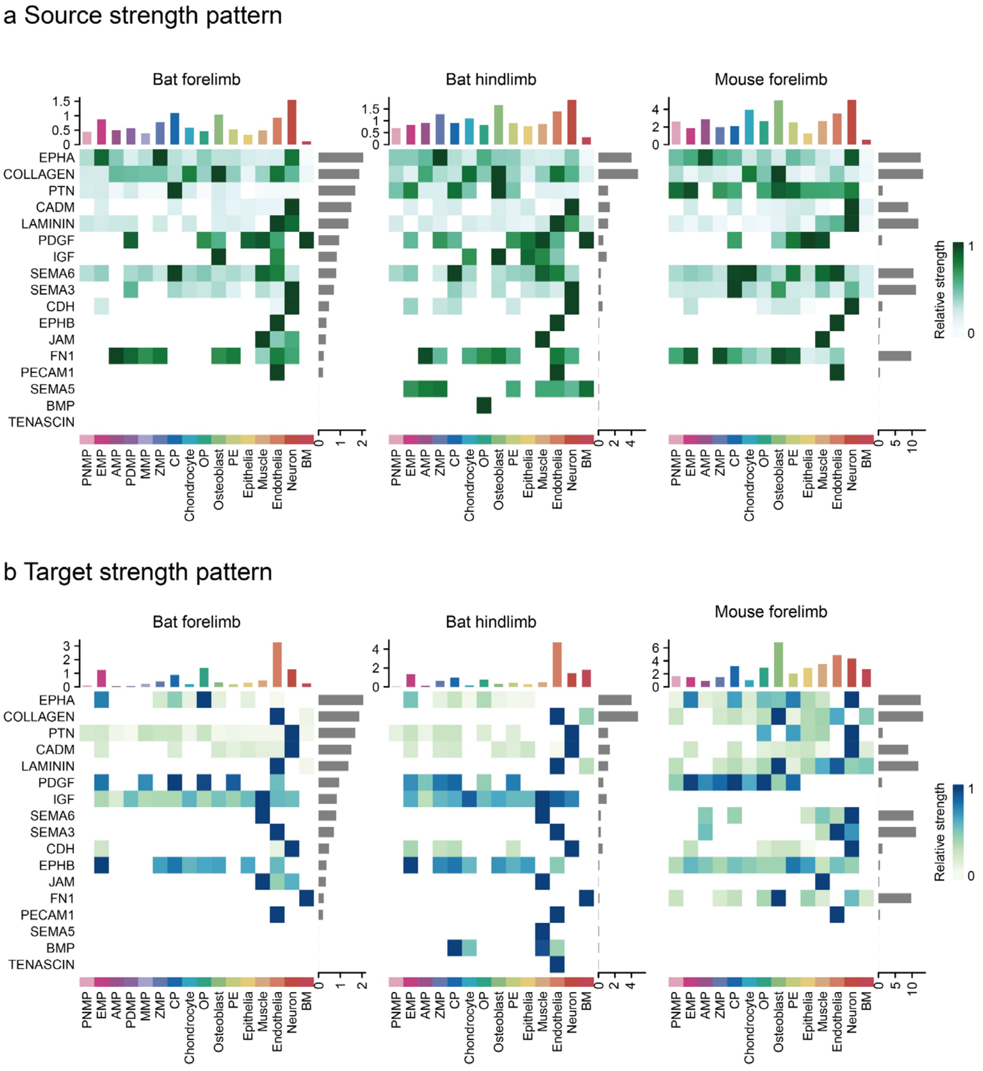

Supplementary Fig. 11. Heatmap of signaling pathways contributing to the incoming and outgoing signaling patterns of each cell population for bat forelimbs, bat hindlimbs, and mouse forelimbs.

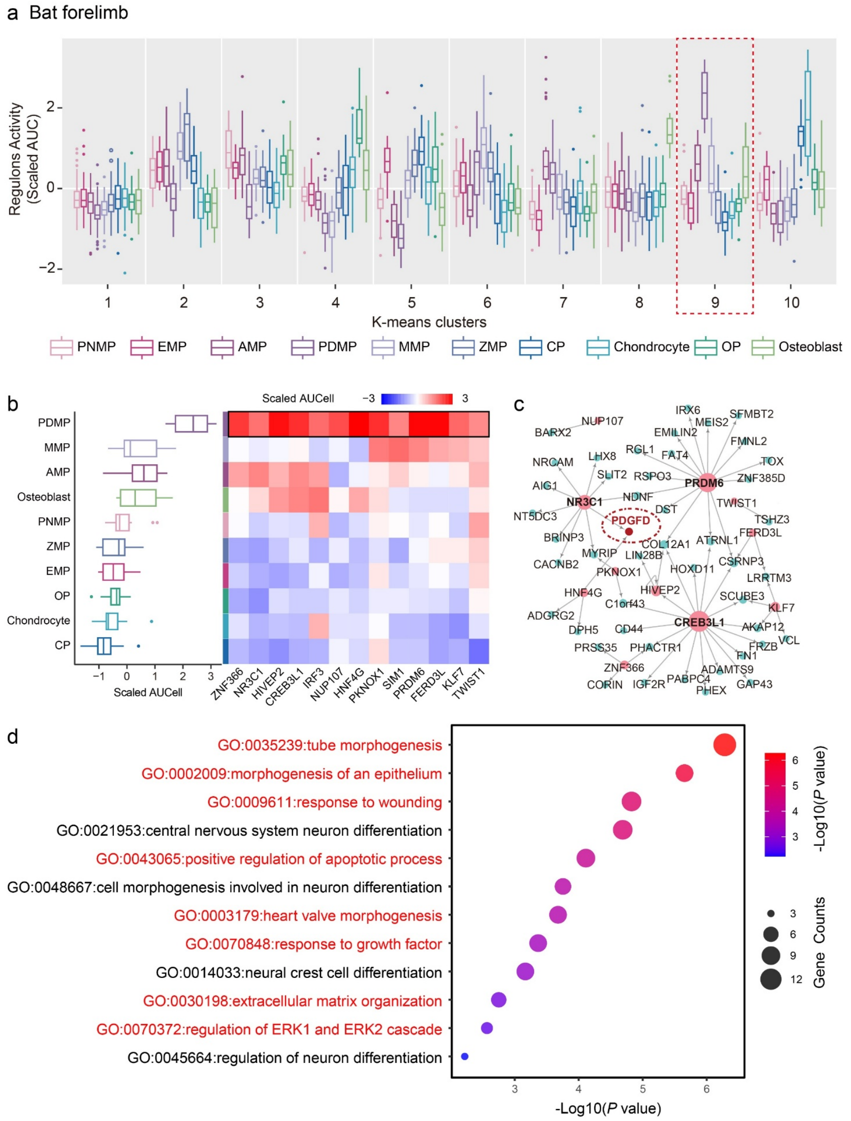

Supplementary Fig. 12. (a) Boxplots display AUCell scores for different clusters in mesenchymal and osteochondral cell populations in bat forelimbs. The box indicates the cluster with TFs that are highly activated in PDMPs. (b) The cluster highlights regulons up-activated in PDMPs compared to other cell populations in the developing bat forelimbs, along with 13 master TFs corresponding to these regulons. (c) The regulation network for 13 master TFs and their TGs differentially expressed between the developing bat forelimbs and hindlimbs. (d) Enriched functional terms for TFs highly activated in PDMPs and their target genes are shown, with the red terms indicating potential contributions to wing membrane development.

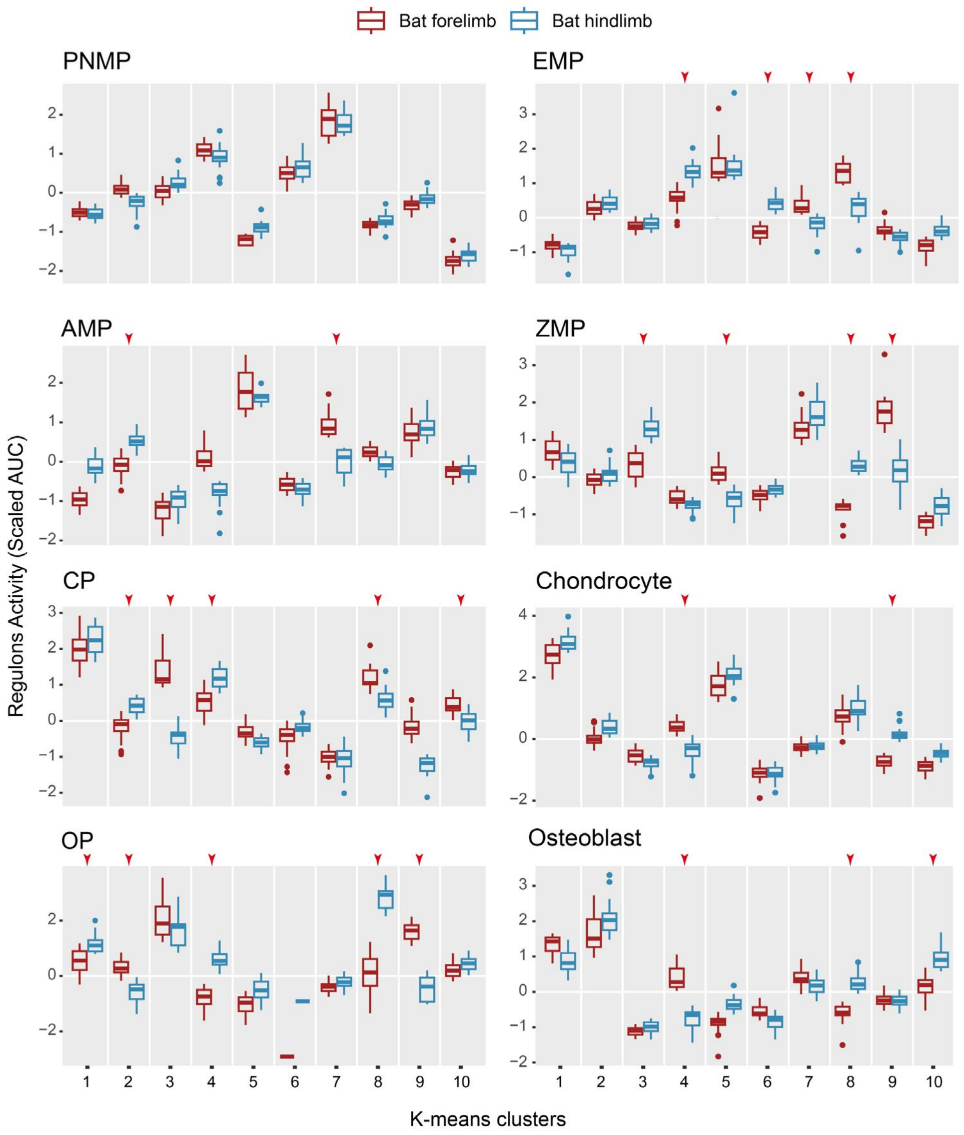

Supplementary Fig. 13. Boxplots illustrate AUCell scores of different clusters in mesenchymal and osteochondral cell populations, comparing developing bat forelimbs and hindlimbs. The arrow-noted clusters contain enriched TFs differentially activated between bat forelimbs and hindlimbs, based on an average difference threshold greater than 0.5 and maximum scores exceeding 0.1 for either forelimbs or hindlimbs.

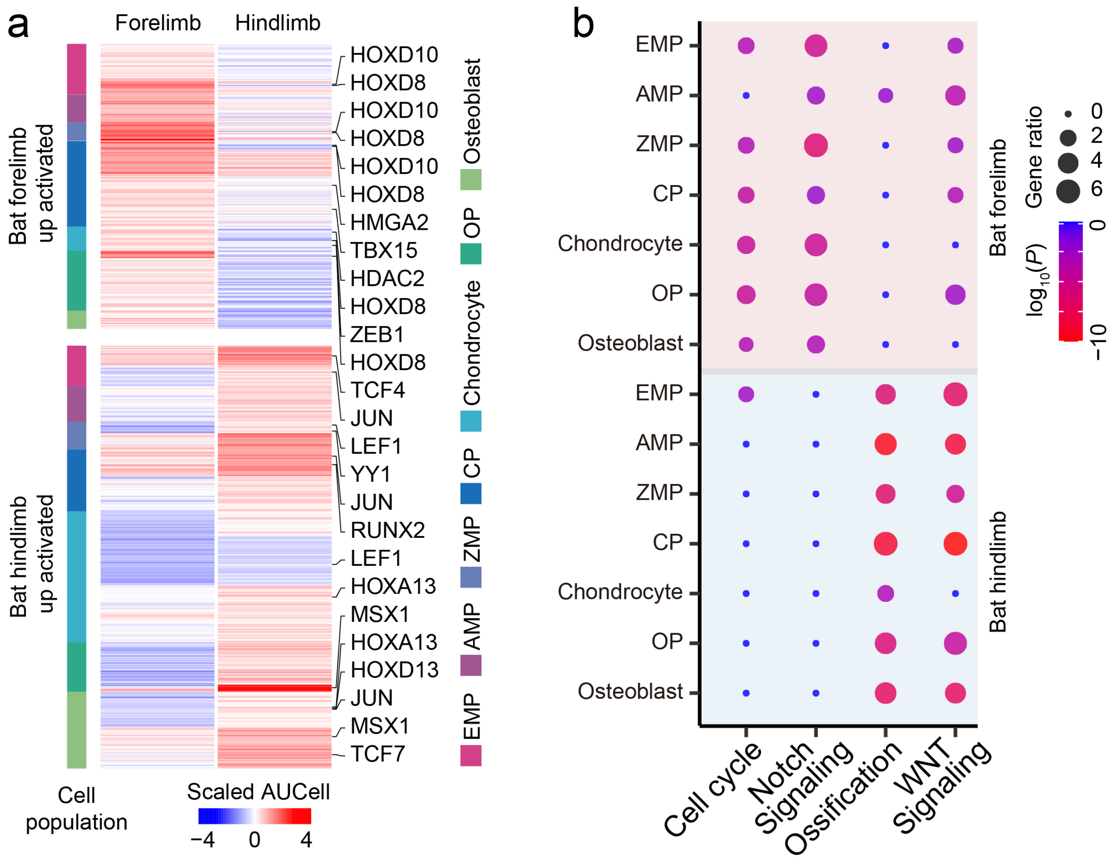

Supplementary Fig. 14. (**a**) Heatmap displaying differentially activated regulons and representative TFs in developing bat forelimbs and hindlimbs. (**b**) Dot plot illustrating enriched pathways for the master TFs and their TGs in developing bat forelimbs and hindlimbs.
