# Supplementary material for: Single-cell expression profiling of bat wing development
Source: Nat Commun. 2025 Jul 18;16:6612. doi: 10.1038/s41467-025-61944-2 (PMC12274464; doi:10.1038/s41467-025-61944-2)
Supplement: Supplementary file 2 — Description of Additional Supplementary File [file 41467_2025_61944_MOESM2_ESM.pdf]

Description of Additional supplementary files

Supplementary Data 1. Digit measurements for three species across different developmental stages.

Supplementary Data 2. Summary of clean scRNA-seq datasets for the developing bat and mouse limbs using SPLiT-seq.

Supplementary Data 3. Barcode information of SPLiT-seq for different samples.

Supplementary Data 4. Summary of clean scRNAseq datasets for the developing bat limbs using 10 × Genomics.

Supplementary Data 5. Summary of bulk RNAseq and ATAC-seq datasets for the developing bat limbs.

Supplementary Data 6. The 13 master TFs and their TGs differentially expressed between the developing bat forelimbs and hindlimbs.

Supplementary Data 7. Differentially activated TFs in bat forelimb and hindlimb for each cell population.

Supplementary Data 8. Top 20 enriched GO terms for differentially expressed genes between the developing bat forelimbs and hindlimbs across different cell clusters.

Supplementary Data 9. Primer and prob sequences for WISH in the developing bat forelimbs and hindlimbs.
