# Supplementary material for: Single-cell expression profiling of bat wing development
Source: Nat Commun. 2025 Jul 18;16:6612. doi: 10.1038/s41467-025-61944-2 (PMC12274464; doi:10.1038/s41467-025-61944-2)
Supplement: Supplementary file 12 — Reporting summary [file 41467_2025_61944_MOESM12_ESM.pdf]

nature portfolio

Corresponding author(s): Zhen Liu

Last updated by author(s): Jun 10, 2025

# Reporting Summary

Nature Portfolio wishes to improve the reproducibility of the work that we publish. This form provides structure for consistency and transparency in reporting. For further information on Nature Portfolio policies, see our Editorial Policies and the Editorial Policy Checklist.

## Statistics

For all statistical analyses, confirm that the following items are present in the figure legend, table legend, main text, or Methods section.

| n/a | Confirmed | |
|---|---|---|
| ☐ | ☒ | The exact sample size (*n*) for each experimental group/condition, given as a discrete number and unit of measurement |
| ☐ | ☒ | A statement on whether measurements were taken from distinct samples or whether the same sample was measured repeatedly |
| ☐ | ☒ | The statistical test(s) used AND whether they are one- or two-sided<br>*Only common tests should be described solely by name; describe more complex techniques in the Methods section.* |
| ☐ | ☒ | A description of all covariates tested |
| ☐ | ☒ | A description of any assumptions or corrections, such as tests of normality and adjustment for multiple comparisons |
| ☐ | ☒ | A full description of the statistical parameters including central tendency (e.g. means) or other basic estimates (e.g. regression coefficient) AND variation (e.g. standard deviation) or associated estimates of uncertainty (e.g. confidence intervals) |
| ☐ | ☒ | For null hypothesis testing, the test statistic (e.g. *F*, *t*, *r*) with confidence intervals, effect sizes, degrees of freedom and *P* value noted<br>*Give P values as exact values whenever suitable.* |
| ☒ | ☐ | For Bayesian analysis, information on the choice of priors and Markov chain Monte Carlo settings |
| ☒ | ☐ | For hierarchical and complex designs, identification of the appropriate level for tests and full reporting of outcomes |
| ☒ | ☐ | Estimates of effect sizes (e.g. Cohen's *d*, Pearson's *r*), indicating how they were calculated |

*Our web collection on statistics for biologists contains articles on many of the points above.*

## Software and code

Policy information about availability of computer code

| | |
|---|---|
| Data collection | No software was used to collect data. |
| Data analysis | dropEst (Version: 0.8.6); STAR (Version: 2.7.1a); Seurat_4.3.0.1; monocle3_1.3.1; scvelo (Version: 0.2.5); cellAlign_0.1.0; CellChat_1.6.1; SCENIC (Version: 0.11.2); Trimmomatic (Version: 0.38); RSEM (Version: 1.3.3); BEDTools (Version 2.30.0)<br>All custom codes are available at GitHub (https://github.com/bettycatherine/Bat-Limb-Dev). |

For manuscripts utilizing custom algorithms or software that are central to the research but not yet described in published literature, software must be made available to editors and reviewers. We strongly encourage code deposition in a community repository (e.g. GitHub). See the Nature Portfolio guidelines for submitting code & software for further information.

## Data

Policy information about availability of data

All manuscripts must include a data availability statement. This statement should provide the following information, where applicable:

- Accession codes, unique identifiers, or web links for publicly available datasets
- A description of any restrictions on data availability
- For clinical datasets or third party data, please ensure that the statement adheres to our policy

All data generated or analyzed during this study are included in the Article and its Supplementary Information. All scRNA-seq and bulk RNA-seq data have been

deposited in the NCBI database (BioProject: all raw data available at PRJNA1062446, single cell processed data available at GSE282269) and in the Genome Sequence Archive at the BIG data center, Chinese Academy of Sciences (accession number: PRJCA022686).

## Research involving human participants, their data, or biological material

Policy information about studies with human participants or human data. See also policy information about sex, gender (identity/presentation), and sexual orientation and race, ethnicity and racism.

| | |
|---|---|
| Reporting on sex and gender | No human participants. |
| Reporting on race, ethnicity, or other socially relevant groupings | No human participants. |
| Population characteristics | No human participants. |
| Recruitment | No human participants. |
| Ethics oversight | No human participants. |

Note that full information on the approval of the study protocol must also be provided in the manuscript.

# Field-specific reporting

Please select the one below that is the best fit for your research. If you are not sure, read the appropriate sections before making your selection.

☐ Life sciences ☐ Behavioural & social sciences ☒ Ecological, evolutionary & environmental sciences

For a reference copy of the document with all sections, see nature.com/documents/nr-reporting-summary-flat.pdf

# Ecological, evolutionary & environmental sciences study design

All studies must disclose on these points even when the disclosure is negative.

| | |
|---|---|
| Study description | Bats are the only true-flight mammals, with wings formed by elongated digits and wing membranes. Despite the uniqueness, the cellular and molecular aspects of bat wing development remains largely unknown. Here, we used single-cell transcriptomic sequencing to map ~39,000 cells from bat (Rhinolophus sinicus) limbs at developmental stages Carnegie stages (CS) 16, 18, and 20. We identified 16 distinct cell populations, including a specific mesenchymal progenitor population (PDGFD+) in bat forelimbs, which may differentiate into the interdigital membrane and promote bone cell proliferation. Developing bat forelimbs exhibited prolonged chondrogenesis and delayed osteogenesis, resulting in more chondrocytes and fewer osteoblasts. The integrative analyses of data from single-cell and bulk RNA sequencing highlighted the crucial roles of Notch signaling activation and WNT/β-catenin signaling suppression in bat forelimb development. Our findings provide a comprehensive single-cell atlas of developing bat limbs, offering insights into the mechanisms underlying bat wing development. |
| Research sample | In order to investigate the phenotype and cellular contributions of the specialization of bat forelimbs, we collected embryonic samples of the eastern bent-winged bat (Miniopterus fuliginosus), the Chinese rufous horseshoe bat (Rhinolophus sinicus), and C57BL/6 mouse. |
| Sampling strategy | For phenotypic analysis, the sampling of the eastern bent-winged bat (Miniopterus fuliginosus) and the Chinese rufous horseshoe bat (Rhinolophus sinicus) covered a series of consecutive developmental stages ranging from Carnegie Stage 16 to 21 (CS16 to CS21), and mice with correspondingly developmental stages from E13.5 to E17.5. We chose three biological samples in each group for experiments.<br>For sequencing and follow-up experiments, we collected embryonic samples of the Chinese rufous horseshoe bat forelimbs and hindlimbs at CS16, CS18, and CS20, and mice with correspondingly developmental stages from E13.5 to E16.5.<br>We chose three biological samples in each group for experiments, and selected two-tailed student's t tests as statistical methods. |
| Data collection | Bai recorded and collected the phenotypic data; Lyu collected scRNA-seq data; Ma collected bulk RNA-seq; Sun, Bai and Jiang recorded and collected the experimental data of each step objectively during every experiment. |
| Timing and spatial scale | Bat embryos were collected in Kunming, Yunnan, China, from April to May during the years 2019 to 2024. |
| Data exclusions | No data were excluded from the analyses. |
| Reproducibility | To verify the reproducibility of our findings, all experiments were repeated three times independently. Tissues from different individuals were used to ensure consistency. All attempts to repeat the experiments were successful, and the results consistently matched the original findings. The results of each replicate were presented in the figures as individual dots. |
| Randomization | Samples for sequencing and experiments were allocated due to developmental stages, with all other conditions randomized. |

| | |
|---|---|
| Blinding | Blinding was not applied in this study. Variation across species is not the main concern of this research. For bioinformatics, reference genome need to be selected based on different species. For experiment, we could distinguish species name from different cell lines. |

Did the study involve field work? ☐ Yes ☒ No

# Reporting for specific materials, systems and methods

We require information from authors about some types of materials, experimental systems and methods used in many studies. Here, indicate whether each material, system or method listed is relevant to your study. If you are not sure if a list item applies to your research, read the appropriate section before selecting a response.

## Materials & experimental systems

| n/a | Involved in the study |
|---|---|
| ☐ | ☒ Antibodies |
| ☐ | ☒ Eukaryotic cell lines |
| ☒ | ☐ Palaeontology and archaeology |
| ☐ | ☒ Animals and other organisms |
| ☒ | ☐ Clinical data |
| ☒ | ☐ Dual use research of concern |
| ☒ | ☐ Plants |

## Methods

| n/a | Involved in the study |
|---|---|
| ☒ | ☐ ChIP-seq |
| ☒ | ☐ Flow cytometry |
| ☒ | ☐ MRI-based neuroimaging |

## Antibodies

| | |
|---|---|
| Antibodies used | The primary antibodies were anti-COL2A1 (1:500, Santa Cruz, Cat. no. sc-52658, Lot. no. D2924), anti-Tarsh Rabbit pAb (anti-ABI3BP) (1:200, Servicebio, Cat. no. GB114912, Lot. no. AC250303013); the secondary antibodies were Goat anti-Mouse IgG (H+L) Highly Cross-Adsorbed Secondary Antibody, Alexa Fluor™ Plus 647 (1:500, Invitrogen, Cat. no. A32728TR, Lot. no. YB367677), Goat anti-Rabbit IgG (H+L) Cross-Adsorbed Secondary Antibody, Cyanine3 (1:500, Invitrogen, Cat. no. A10520, Lot. no. 2738238). |
| Validation | From manufacturer websites:<br>anti-COL2A1: https://www.scbt.com/zh/p/col2a1-antibody-m2139#citations<br>anti-ABI3BP: https://www.servicebio.cn/goodsdetail?id=13231<br>Goat anti-Mouse IgG (H+L) Highly Cross-Adsorbed Secondary Antibody, Alexa Fluor™ Plus 647: https://www.thermofisher.cn/cn/zh/antibody/product/Goat-anti-Mouse-IgG-H-L-Highly-Cross-Adsorbed-Secondary-Antibody-Polyclonal/A32728TR<br>Goat anti-Rabbit IgG (H+L) Cross-Adsorbed Secondary Antibody, Cyanine3: https://www.thermofisher.cn/cn/zh/antibody/product/Goat-anti-Rabbit-IgG-H-L-Cross-Adsorbed-Secondary-Antibody-Polyclonal/A10520 |

## Eukaryotic cell lines

Policy information about cell lines and Sex and Gender in Research

| | |
|---|---|
| Cell line source(s) | We used four cell lines from three species, including human (HEK 293T cells), laboratory mouse (MC3T3-E1) and the Chinese rufous horseshoe bat (embryonic forelimb cells and embryonic fibroblasts). We did not consider the effect of sex of cell lines in this study. |
| Authentication | None of the cell lines used were authenticated. |
| Mycoplasma contamination | All cell lines tested negative for mycoplasma contamination. |
| Commonly misidentified lines<br>(See ICLAC register) | None. |

## Animals and other research organisms

Policy information about studies involving animals; ARRIVE guidelines recommended for reporting animal research, and Sex and Gender in Research

| | |
|---|---|
| Laboratory animals | Laboratory mouse(C57BL/6), developmental stages from E13.5 to E17.5. |
| Wild animals | The bats were captured from caves, including the Chinese rufous horseshoe bat (Rhinolophus sinicus) and the eastern bent-winged bat (Miniopterus fuliginosus). We euthanized adult females using an overdose of isoflurane inhalation immediately after capture to obtain embryos. |
| Reporting on sex | We only collected female bats/mice to obtain embryos. We did not consider the effect of embryonic sex in this study. |

Field-collected samples | The bat samples were obtained from the field.

Ethics oversight | All animals procedures were approved by the Ethics Committee of the Kunming Institute of Zoology, CAS.

Note that full information on the approval of the study protocol must also be provided in the manuscript.

## Plants

Seed stocks | No plants in this study.

Novel plant genotypes | No plants in this study.

Authentication | No plants in this study.
